# Attenuated* Legionella pneumophila* Survives for a Long Period in an Environmental Water Site

**DOI:** 10.1155/2019/8601346

**Published:** 2019-07-03

**Authors:** Takashi Nishida, Natsuko Nakagawa, Kenta Watanabe, Takashi Shimizu, Masahisa Watarai

**Affiliations:** The United Graduate School of Veterinary Science, Yamaguchi University, 1677-1 Yoshida, Yamaguchi 753-8515, Japan

## Abstract

*Legionella pneumophila* is known as a human pathogen and is ubiquitous in natural and artificial aquatic environments. Many studies have revealed the virulence traits of* L. pneumophila* using clinical strains and a number of studies for characterizing environmental strains are also reported. However, the association between the virulence and survivability in the environment is unclear. In the present study,* L. pneumophila* was isolated from environmental water sites (Ashiyu foot spa, water fountain, and public bath), and the serogroups of isolated strains were determined by serological tests. Isolated strains were found to belong to serogroups SG1, SG2, SG3, SG4, SG5, SG8, SG9, and SG13. Untypeable strains were also obtained. Isolated strains were used for intracellular growth assay in a human monocytic cell line, THP-1. Among these strains, only an untypeable strain, named AY3, failed to replicate in THP-1. In addition, AY3 was maintained for a long period in an environmental water site, Ashiyu foot spa 2. Further, we compared the characteristics of several strains isolated from Ashiyu foot spa 2 and a clinical strain, Togus-1. AY3 failed to replicate in THP-1 cells but replicated in an amoeba model,* Dictyostelium discoideum*. Compared with Togus-1, the culturable cell number of environmental strains under stress conditions was higher. Moreover, biofilm formation was assessed, and AY3 showed the same degree of biofilm formation as Togus-1. Biofilm formation, replication in amoebae, and resistance against stress factors would explain the predominance of AY3 at one environmental site. Although the mechanism underlying the difference in the ability of AY3 to replicate in THP-1 cells or amoebae is still unclear, AY3 may abandon the ability to replicate in THP-1 cells to survive in one environment for a long period. Understanding the mechanisms of* L. pneumophila* in replication within different hosts should help in the control of Legionnaires' disease, but further study is necessary.

## 1. Introduction

*Legionella pneumophila* is a facultative intracellular gram-negative bacterium and a known human pathogen [1]. This bacterium replicates in alveolar macrophages after infection in humans. Many studies have revealed a number of factors which contribute to the virulence of* L. pneumophila* using clinical strains [2–4].* L. pneumophila* is ubiquitous in natural and artificial aquatic environments [5, 6] and infects humans by inhalation of* Legionella*-containing aerosols from the environmental water sites. A number of studies for characterizing environmental strains are also reported [7–10].* L. pneumophila *is divided into 15 serogroups (SG1-SG15), and SG1 is the predominant serogroup in the identified clinical cases; however, the ratio of SG1 in environmental isolates is lower (approximately from 20% to 40%) compared with the ratio of SG1 in clinical isolates (approximately 90%) [1, 11–15]. SG1 may be able to more efficiently infect human or exhibit higher virulence compared with the other SGs. The other possibility is that the survivability of* L. pneumophila* in the environment differs among SGs. In the environment, there are many stress factors, such as temperature, pH, oxidative stress, chlorine, and protists such as amoebae which may influence the survival of* L. pneumophila *[6, 16–18]. However, the association between the virulence and survivability in the environment is unclear. Therefore, in this study, we isolated* L. pneumophila* from environmental water sites and investigated the characteristics of isolated strains.

## 2. Materials and Methods

### 2.1. Bacteria and Culture Conditions

*L. pneumophila* strains were maintained as frozen glycerol stocks and cultured at 37°C on N-(2-acetamido)-2-aminoethanesulfonic acid-buffered charcoal yeast extract agar (BCYE) or in the same medium without agar and charcoal (AYE) [19]. A clinical strain of* L. pneumophila*, Togus-1 GTC 00746 (ATCC 33154), was obtained from the National BioResouce Project of the Ministry of Education, Culture, Sports, Science, and Technology, Japan (http://www.nbrp.jp/).

### 2.2. Isolation of L. pneumophila

One hundred milliliters of sample was collected from each site in sterile bottles and centrifuged at 3,000 ×* g* for 30 min. The deposits were resuspended in 1 mL phosphate-buffered saline (PBS) as concentrates. Concentrated samples were heated at 50°C for 30 min and spread onto the surface of GVPC. GVPC is BCYE supplemented with glycine (Wako, Osaka, Japan, 3 mg/mL), vancomycin HCl (Wako, 1 *μ*g/mL), polymyxin B (Wako, 80 IU/mL), and sulfate cycloheximide (Wako, 80 *μ*g/mL). Plates were incubated at 37°C and inspected daily. Isolated bacteria were grown on BCYE or BCYE without cysteine at 37°C. Cysteine auxotrophic bacteria were used for PCR analysis.

### 2.3. PCR Analysis, Serotyping, and Sequence-Based Typing

To confirm whether cysteine auxotrophic bacteria were* L. pneumophila* or not, the presence of the* L. pneumophila-*specific gene,* mip* [20], was tested by PCR using mip1/mip2 primers (mip1: 5′-GGTGACTGCGGCTGTTATGG-3′ and mip2: 5′- GGCCAATAGGTCCGCCAACG-3′). After denaturation of the bacterial chromosomal DNA template at 95°C for 5 min, 35 cycles of PCR amplification were performed using Tks Gflex DNA Polymerase (Takara, Tokyo, Japan). The serogroups of PCR-positive bacteria were determined based on their reactions during immunoagglutination serotyping with* Legionella* immune sera (Denka Seiken, Tokyo, Japan).

Sequence-based typing was performed according to the protocol (version 5.0) developed by the European Working Group for* Legionella* Infections [21, 22]. Briefly, seven genes (*flaA*,* pilE*,* asd*,* mip*,* mompS*,* proA*, and* neuA*) of* L. pneumophila* were amplified by PCR. The sequences of amplicons were determined using a BigDye Terminator v3.1 Cycle Sequencing Kit and an ABI3031 Genetic Analyzer (Thermo Fisher Scientific Inc., Waltham, MA, USA). The sequences were compared with previously assigned allele numbers using the database www.hpa-bioinformatics.org.uk/legionella/legionella_sbt/php/sbt_homepage.php. The sequence type is represented by a number (e.g., ST1) depending on the combination of the allele numbers of seven genes (e.g., 1, 4, 3, 1, 1, 1, and 1).

### 2.4. Cell Lines and Culture Conditions

A human monocytic cell line, THP-1, was grown at 37°C and 5% CO_2_ in RPMI 1640 medium (Sigma-Aldrich, MO, USA), containing 10% heat-inactivated FBS (Biowest, Paris, France). THP-1 cells were differentiated with 100 nM phorbol 12-myristate 13-acetate (PMA, Sigma) 48 h prior to use.* Dictyostelium discoideum* Ax-2 was cultured in shaken flasks at 25°C using HL-5 medium (5 mg/mL of yeast extract, 10 mg/mL of glucose, 10 mg/mL of proteose peptone, 0.64 mg/mL of Na_2_HPO_4_, and 0.48 mg/mL of KH_2_PO_4_ [23]).

### 2.5. Intracellular Growth Assays

Intracellular growth assay was performed as described previously [24, 25] with a slight modification. In brief, bacteria were added to a monolayer of THP-1 cells in 48-well tissue culture dishes at a multiplicity of infection (MOI) of 1. These plates were centrifuged for 10 min at 900 ×*g* and incubated for 1 h at 37°C. Then medium was changed to gentamicin-containing (50 *μ*g/mL) medium and incubated for 1 h at 37°C. To measure the intracellular growth, the cells were incubated in fresh medium at 37°C for a particular time and washed with RPMI 1640 medium, followed by lysis with cold distilled water. Colony forming units (CFU) counts were determined by serial dilution on BCYE.

Bacteria were added to* D. discoideum* Ax-2 in 48-well tissue culture dishes at an MOI of 1. These plates were centrifuged for 5 min at 250 ×*g* and incubated for 1 h at 25°C. Then medium was changed to gentamicin-containing (50 *μ*g/mL) medium and incubated for 1 h at 37°C. To quantify intracellular growth, the cells were incubated in fresh medium at 25°C for a designated amount of time and then lysed with 0.02% saponin (Wako). CFU counts were determined by serial dilution on BCYE.

### 2.6. Heat Resistance, Acid Resistance, and Oxidative Stress Resistance Assay

To examine heat resistance, the medium of bacterial cultures was removed after centrifugation for 5 min at 5000×*g* and the bacterial pellet was resuspended in PBS. This step was repeated two times. Suspensions were subsequently heat-treated for 30 min at 55°C. To examine acid stress resistance, bacterial pellets were resuspended in 0.2 M HCl-KCl buffer (pH 2.2) and incubated for 6 h at room temperature. After incubation, CFU counts were determined using serial dilution on BCYE. The percentage of culturable bacteria was calculated by dividing the CFU of treated bacteria by the CFU of untreated bacteria.

To examine oxidative stress resistance, bacterial cultures were diluted 100 times by AYE with or without 2 mM hydrogen peroxide and incubated for 6 h at 37°C. After incubation, CFU counts were determined using serial dilution on BCYE. The relative increase of bacteria was calculated by dividing the CFU of treated bacteria by the CFU of untreated bacteria.

### 2.7. Chlorine Resistance Assay

Sodium hypochlorite solution (Wako) was diluted in PBS to prepare experimental solutions. The concentration of free residual chlorine was determined by DPD method using chlorine comparators (SIBATA, Saitama, Japan) and adjusted to 0.1 ppm. The bacteria concentration was adjusted to approximately 10^7^ CFU/mL in PBS, and 100 *μ*L of suspension was added to 10 mL of 0.1 ppm experimental solution. After exposure to chlorine at room temperature for 1 min and 3 min, 1 mL of this solution was added to a sterile tube containing 15 *μ*L of 0.3 M sodium thiosulfate solution to neutralize the residual chlorine. CFU counts were determined using serial dilution on BCYE. The percentage of culturable bacteria was calculated by dividing the CFU of treated bacteria by the CFU of untreated bacteria.

### 2.8. Biofilm Formation Assay

Biofilms were established as previously described [26]. In brief, bacteria were diluted in 10% AYE solution to approximately 10^7^ CFU/mL, and 100 *μ*L of this solution was inoculated into 96-well polystyrene microtiter plates. After incubation for 24 h at 37°C, culture medium was changed to 200 *μ*L AYE. Cultures were incubated at 37°C for 1 to 4 days. To quantify biofilm mass, planktonic bacteria were discarded, and 200 *μ*L methanol was added to each well and incubated for 15 min to fix surface-attached cells. Biofilms were stained for 5 min at room temperature with 1% crystal violet solution. Subsequently, the wells were washed three times with distilled water, and the dye was solubilized in 33% glacial acetic acid. The resulting solution was finally assayed to determine the optical density at 570 nm with the microplate reader.

### 2.9. Statistical Analyses

Statistical analyses were performed using Student's* t*-test with a Bonferroni correction. Statistically significant differences between groups were accepted at* P *< 0.05 or* P* < 0.01. Data are presented as the average based on triplicate samples (intracellular growth assay and biofilm formation assay) or duplicate samples (resistance assay) of three identical experiments done in different times, and the error bars shown in the figures represent standard deviations.

## 3. Results

### 3.1. Untypeable Strains Are Maintained in an Ashiyu Foot Spa

Samples were collected from environmental water sites in Yamaguchi Prefecture and Kochi Prefecture, Japan in 2016.* L. pneumophila* strains were isolated from six sites (Table 1). These isolates belonged to serogroups SG1, SG2, SG3, SG4, SG5, SG8, SG9, and SG13. Untypeable strains (UTs) were also obtained. One strain, which belonged to a different SG, was also picked (Table 1), and bacterial loads in THP-1 cells of these strains were examined 2 h and 48 h after infection. As a result, among these 15 strains, only AY3 did not replicate in THP-1 (Table 1). In previous reports, isolated* L. pneumophila* strains from the environment could replicate in mammalian macrophage cell lines [7, 9, 10]. In addition, the number of UTs was dominant among strains isolated from Ashiyu foot spa 2. Therefore, we focused on isolates from Ashiyu foot spa 2.

**Figure 1:**
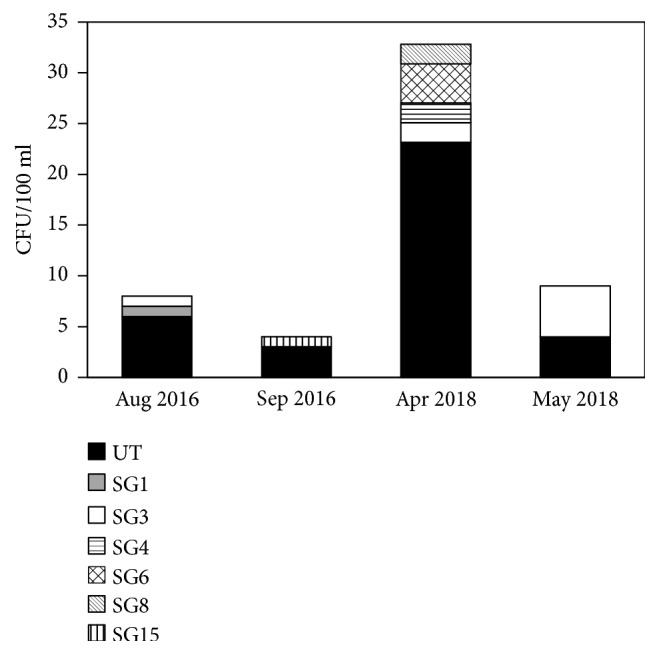
The serogroups of* L. pneumophila* isolated from Ashiyu foot spa 2 and the numbers of each serogroup.* L. pneumophila* isolation was performed in August and September 2016 and April and May 2018. The serogroups were determined based on the reaction during the immunoagglutination serotyping with* Legionella* immune sera.

To determine whether the UTs in Ashiyu foot spa 2 were maintained for a long period, we collected samples from the same place in August and September 2016 and April and May 2018. As a result,* L. pneumophila* strains which belonged to SG1, SG3, SG4, SG6, SG8, SG15, and UT were isolated (Figure 1). The ratio of UT in each sample ranged from 44% to 85%. To determine whether UTs were derived from a single strain or not, we randomly picked 8 UTs and classified these strains by sequence-based typing. The sequence type of these all strains and AY3 was ST1319. These 8 UTs failed to grow in THP-1 cell (data not shown). These results indicate that the UTs in Ashiyu foot spa 2 were derived from the same original strain, and AY3 have dominantly survived for a long time in the environmental water site.

### 3.2. Untypeable Strain AY3 Fails to Replicate in THP-1

**Figure 2:**
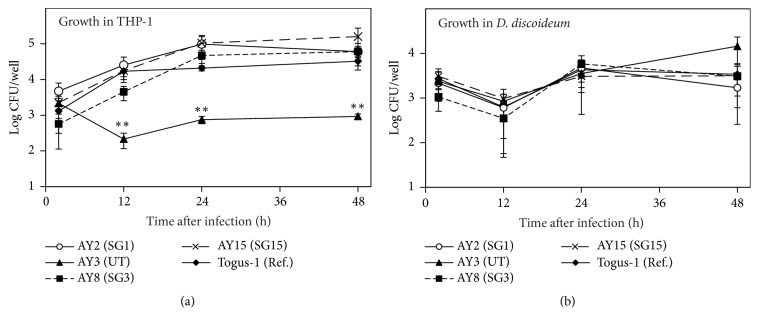
Intracellular growth of environmental strains from Ashiyu foot spa 2. (a) THP-1 cells were infected with* L. pneumophila* strains at an MOI of 1 for 1 h. Then medium was changed to gentamicin-containing (50 *μ*g/mL) medium and incubated for 1 h at 37°C. After incubation for particular time, the infected cells were washed with RPMI 1640 medium, followed by lysis with cold distilled water. CFU counts were determined by serial dilution on BCYE. (b)* D. discoideum* was infected with* L. pneumophila* strains at an MOI of 1 for 1 h. Then medium was changed to gentamicin-containing (50 *μ*g/mL) medium and incubated for 1 h at 37°C. After incubation for particular time, the infected cells lysed with 0.02% saponin. CFU counts were determined by serial dilution on BCYE. All values represent the average and the standard deviation for three identical experiments. Statistically significant differences compared with Togus-1 are indicated by asterisks (*∗∗*,* P* < 0.01).

As the bacterial load of AY3 did not increase 48 h after infection in THP-1 cells, we evaluated intracellular replication in detail at 2, 12, 24, and 48 h after infection using environmental strains from Ashiyu foot spa 2 in 2016 (Figure 2(a)). AY2, AY8, and AY15 showed the same growth rate compared with the clinical strain Togus-1. In contrast, the number of AY3 decreased 12 h after infection, and no replication was observed. Because* L. pneumophila* usually replicates in protistan hosts such as amoebae in the environment [17, 27], we evaluated intracellular growth in* D. discoideum*, which is an amoeba infection model of* L. pneumophila* (Figure 2(b)). As a result, all strains, including AY3, increased to the same level as the clinical strain Togus-1. These results imply that AY3 abandons the growth ability in THP-1 cells but still maintains the ability in protistan hosts in the environment.

### 3.3. The Environmental Strains Show Resistance against Several Stress Conditions

**Figure 3:**
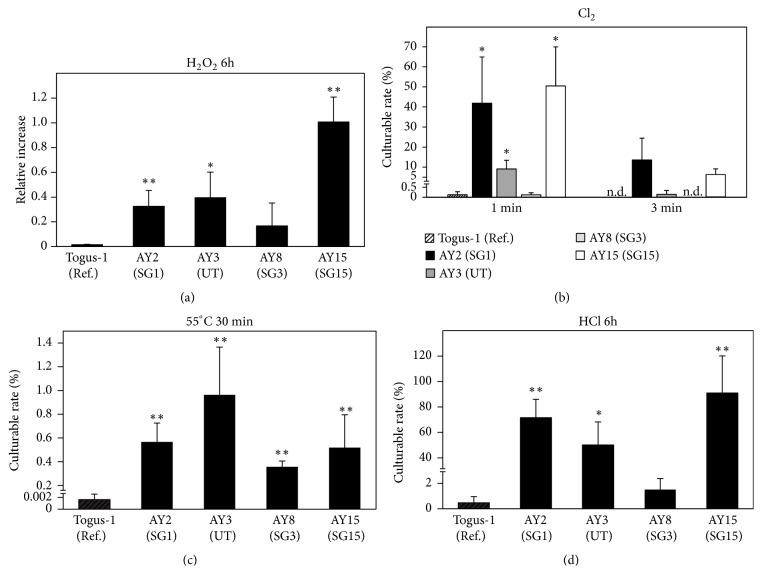
Resistance of environmental strains from Ashiyu foot spa 2 against stress factors. (a) Each strain was incubated for 6 h at 37°C with or without 2 mM hydrogen peroxide (H_2_O_2_). CFU counts were determined using serial dilution on BCYE. The relative increase was calculated by dividing the CFU of culture with H_2_O_2_ by the CFU of culture without H_2_O_2_. (b) Each strain was exposed to 0.1 ppm chlorine (Cl_2_) at room temperature for 1 min and 3 min. After neutralization, CFU counts were determined using serial dilution on BCYE. The percentage of culturable bacteria was calculated by dividing the CFU of treated bacteria by the CFU of untreated bacteria (n.d.: not detected). (c) Each strain was heat-treated for 30 min at 55°C. CFU counts were determined using serial dilution on BCYE. The percentage of culturable bacteria was calculated by dividing the CFU of treated bacteria by the CFU of untreated bacteria. (d) Each strain was suspended by 0.2 M HCl-KCl buffer (pH2.2) and incubated for 6 h at room temperature. CFU counts were determined using serial dilution on BCYE. The percentage of culturable bacteria was calculated by dividing the CFU of treated bacteria by the CFU of untreated bacteria. All values represent the average and the standard deviation for three identical experiments. Statistically significant differences compared with Togus-1 are indicated by asterisks (*∗*,* P* < 0.05, and *∗∗*,* P* < 0.01).

In the environment, there are many stress factors, such as temperature, pH, oxidative stress, and chlorine, which may influence the survival of* L. pneumophila*. Therefore, we assumed that AY3 have resistance against these stress factors because this strain was maintained for a long period in Ashiyu foot spa 2 (Figure 1). The resistance against oxidative stress, chlorine, high temperature, or pH was assessed. Compared with the clinical strain, Togus-1, environmental strains such as AY2, AY3, and AY15 relatively increased in the media containing hydrogen peroxide, and the rate of culturable cells of AY2, AY3, and AY15 was higher when treated with chlorine or HCl (Figures 3(a), 3(b), and 3(d)). Further, the rate of culturable cell of all environmental strains was higher than that of Togus-1 when incubated at 55°C (Figure 3(c)). However, no difference was observed between AY3 and the other three environmental strains.

### 3.4. Biofilm Was Formed by Untypeable Strain AY3

**Figure 4:**
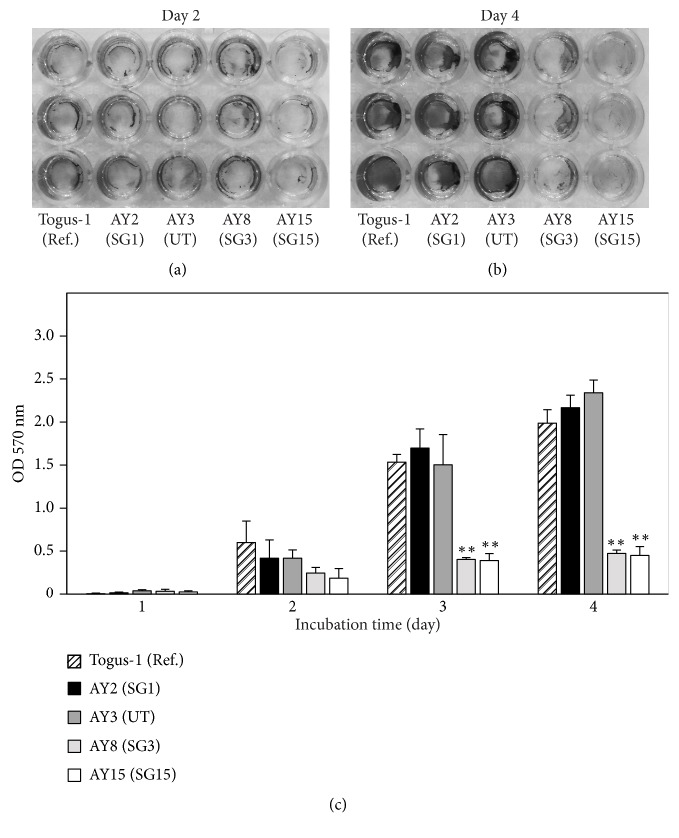
Biofilm formation of environmental strains from Ashiyu foot spa 2. (a), (b) Each strain was inoculated into 96-well plates. After 2-day (a) or 4-day (b) incubation, biofilm mass was stained with crystal violet solution. (c) To quantify biofilm mass, after staining, the dye was solubilized in 33% glacial acetic acid. The optical density at 570 nm of the resulting solution was determined with the microplate reader. All values represent the average and the standard deviation for three identical experiments. Statistically significant differences compared with Togus-1 are indicated by asterisks (*∗∗*,* P* < 0.01).

In aquatic environments, biofilms have been recognized as an important factor of survival and proliferation of* L. pneumophila* [8, 28]. Therefore, we assessed the biofilm formation of environmental strains (Figure 4). At day 2, biofilm was not formed (Figure 4(a)). At days 3 and 4, biofilm was formed by AY2, AY3, and Togus-1 (Figures 4(b) and 4(c)). This result indicates that AY2 and AY3 can form biofilm at the same degree as Togus-1.

## 4. Discussion

Cooling towers and building water systems are reported as the major infectious sources of* L. pneumophila* [29]. In addition, hot spring bathing facilities are also major infectious sources in Japan [14, 30]. To reduce the risk of* L. pneumophila* infection, keeping these facilities clean is important. In Japan, hot spring bathing facilities are required to be cleaned at regular intervals and to be disinfected with chlorine. However, patients of Legionnaires' disease are increasing every year [14]. Therefore, understanding the ecology of* L. pneumophila *in the environment is required to control Legionnaires' disease. In the present study, we isolated* L. pneumophila* from environmental water sites and the properties of isolated strains were analyzed. In Ashiyu foot spa 2, AY3 was maintained dominantly for a long period (Figure 1). In addition, biofilm formation by AY3 was observed (Figure 4), and AY3 had the ability to replicate in amoeba (Figure 2(b)). In the environment,* L. pneumophila* is found in biofilms and replicates in amoebae [8, 28]. Because bacteria are protected from external stresses in biofilms, excluding* L. pneumophila* from artificial environments such as bathing facilities becomes difficult [31, 32]. From this perspective, the long-term predominance of AY3 at the same environmental conditions is thought to be attributed to its abilities of biofilm formation and replication in amoebae. Further, the rate of culturable cell of AY3 under each stress condition was higher compared with the clinical strain, Togus-1 (Figure 3). In the environment,* L. pneumophila* is exposed to many stress factors which may influence its survival. Therefore, resistance against stress factors also may explain the predominance of AY3. However, other environmental strains also replicated in amoeba and showed the same level of the culturable bacteria under stress conditions compared with AY3. Furthermore, biofilm was also formed by AY2. These results suggest that these environmental strains also possess the ability to survive for a long period if the environment permits. The mechanism of environmental strains to survive for a long period in the environment is still unclear. Further study is required to clarify the mechanism.

*L. pneumophila* is divided into 15 serogroups, but UTs are sometimes reported [8, 14]. The genes which determine the serogroup have not yet been revealed, and the association of these genes with the ability to replicate in macrophages is also unclear. In this study, AY3 (UT) failed to replicate in THP-1 cells (Figure 2(a)). However, a UT was also isolated from another environmental water site, and this strain (K6) replicated in THP-1 cells (Table 1). In addition, clinical strains are sometimes reported as UTs; therefore, there would not be an association between the ability to replicate in macrophages and serogroups; AY3 would abandon this ability independently of serogroup-related genes.

Intracellular replication is one of the pathogenic factors of* L. pneumophila*. Therefore, the dominant strain, AY3, may be attenuated because the growth of AY3 in THP-1 cells was lower compared with other strains (Figure 2(a)). On the other hand, AY3 replicated in* D. discoideum* (Figure 2(b)), which has been used as the amoeba host model of* L. pneumophila*. The mechanisms of intracellular replication in amoebae and macrophages are usually closely linked, and mutants defective for growth in macrophages also show defective growth in amoebae [33, 34]. These facts imply that AY3 have a unique mechanism which is necessary to grow only in amoebae but not in THP-1 cells. In addition, the growth condition of AY3 was restricted because this strain was maintained dominantly for a long period in the same environmental water site (Figure 1). It was reported that restricting* L. pneumophila* to replicate in macrophages caused mutations that improved its replication in macrophages and decreased its replication in amoebae [35]. Therefore, restricting the surviving conditions of AY3 to the same environment may provide selective pressure that modifies the traits of this strain to acquire a unique mechanism.

In this work, we focused on environmental strains isolated from an Ashiyu foot spa. In foot spa 2, AY3 was maintained predominantly for a long period. This strain lacked the ability to replicate in THP-1 cells but replicated in amoebae. Although the detailed mechanism to lose growth ability in THP-1 cells is unknown, AY3 may abandon the ability of replication in THP-1 cells to survive in one environment for a long period. Understanding the mechanisms of* L. pneumophila* replication within different hosts should be helpful to control Legionnaires' disease, but further study is necessary.

## Figures and Tables

**Table 1:** Characteristics of *L. pneumophila* isolates.

Place	SG	Strain	*∗*Growth in THP-1
Ashiyu foot spa 1	3	AY1	+
Ashiyu foot spa 2	1	AY2	++
	3	AY8	++
	UT	AY3	-
Water fountain 1	1	AY10	++
Water fountain 2	1	K19	++
	5	K11	++
	13	K27	+
Public bath 1	3	AY17	++
Public bath 2	2	K3	++
	4	K7	+
	8	K8	+
	9	K1	+
	UT	K6	+

*∗* −; less than 1 fold increase, +; 3-10 fold increase, ++; more than 10 fold increase

## Data Availability

The data used to support the findings of this study are available from the corresponding author upon request.

## References

[1] Cunha B. A., Burillo A., Bouza E. (2016). Legionnaires' disease. *The Lancet*.

[2] Andrews H. L., Vogel J. P., Isberg R. R. (1998). Identification of linked Legionella pneumophila genes essential for intracellular growth and evasion of the endocytic pathway. *Infection and Immunity*.

[3] Al-Khodor S., Kalachikov S., Morozova I., Price C. T., Abu Kwaik Y. (2009). The PmrA/PmrB two-component system of legionella pneumophila is a global regulator required for intracellular replication within macrophages and protozoa. *Infection and Immunity*.

[4] Fuche F., Vianney A., Andrea C., Doublet P., Gilbert C., Parkinson J. S. (2015). Functional type 1 secretion system involved in legionella pneumophila virulence. *Journal of Bacteriology*.

[5] Fliermans C. B., Cherry W. B., Orrison L. H. (1981). Ecological distribution of Legionella pneumophila. *Applied and Enbironmental Microbiology*.

[6] Declerck P., Behets J., van Hoef V., Ollevier F. (2007). Detection of Legionella spp. and some of their amoeba hosts in floating biofilms from anthropogenic and natural aquatic environments. *Water Research*.

[7] Garcia-Nuñez M., Pedro-Botet M. L., Ragull S. (2009). Cytopathogenicity and molecular subtyping of Legionella pneumophila environmental isolates from 17 hospitals. *Epidemiology and Infection*.

[8] Chaabna Z., Forey F., Reyrolle M. (2013). Molecular diversity and high virulence of Legionella pneumophila strains isolated from biofilms developed within a warm spring of a thermal spa. *BMC Microbiology*.

[9] Tachibana M., Nakamoto M., Kimura Y., Shimizu T., Watarai M. (2013). Characterization of *Legionella pneumophila* isolated from environmental water and ashiyu foot spa. *BioMed Research International*.

[10] Arslan-Aydoğdu E. Ö., Kimiran A. (2018). An investigation of virulence factors of Legionella pneumophila environmental isolates. *Brazilian Journal of Microbiology*.

[11] Yu V. L., Plouffe J. F., Pastoris M. C. (2002). Distribution of legionella species and serogroups isolated by culture in patients with sporadic community-acquired legionellosis: An international collaborative survey. *The Journal of Infectious Diseases*.

[12] Doleans A., Aurell H., Reyrolle M. (2004). Clinical and environmental distributions of legionella strains in france are different. *Journal of Clinical Microbiology*.

[13] Harrison T., Doshi N., Fry N., Joseph C. (2007). Comparison of clinical and environmental isolates of Legionella pneumophila obtained in the UK over 19 years. *Clinical Microbiology and Infection*.

[14] Amemura-Maekawa J., Kura F., Chida K. (2018). Legionella pneumophila and other *legionella* species isolated from legionellosis patients in japan between 2008 and 2016. *Clinical Microbiology and Infection*.

[15] Laganà P., Facciolà A., Palermo R., Delia S. (2019). Environmental surveillance of legionellosis within an italian university hospital—results of 15 years of analysis. *International Journal of Environmental Research and Public Health*.

[16] Casini B., Aquino F., Totaro M. (2017). Application of hydrogen peroxide as an innovative method of treatment for legionella control in a hospital water network. *Pathogens*.

[17] Rowbotham T. J. (1980). Preliminary report on the pathogenicity of Legionella pneumophila for freshwater and soil amoebae.. *Journal of Clinical Pathology*.

[18] Storey M. V., Winiecka-Krusnell J., Ashbolt N. J., Stenström T.-A. (2004). The efficacy of heat and chlorine treatment against thermotolerant Acanthamoebae and Legionellae. *Scandinavian Journal of Infectious Diseases*.

[19] Nishida T., Watanabe K., Tachibana M., Shimizu T., Watarai M. (2017). Characterization of the cryptic plasmid pOfk55 from Legionella pneumophila and construction of a pOfk55-derived shuttle vector. *Plasmid*.

[20] Ratcliff R. M., Slavin M. A., Sangster N., Doyle R. M., Seymour J. F., Lanser J. A. (2003). Legionella pneumophila mip gene sequencing to investigate a cluster of pneumonia cases. *Pathology*.

[21] Gaia V., Fry N. K., Afshar B. (2005). Consensus sequence-based scheme for epidemiological typing of clinical and environmental isolates of legionella pneumophila. *Journal of Clinical Microbiology*.

[22] Ratzow S., Gaia V., Helbig J. H., Fry N. K., Luck P. C. (2007). Addition of neuA, the gene encoding n-acylneuraminate cytidylyl transferase, increases the discriminatory ability of the consensus sequence-based scheme for typing legionella pneumophila Serogroup 1 Strains. *Journal of Clinical Microbiology*.

[23] Hagele S., Kohler R., Merkert H., Schleicher M., Hacker J., Steinert M. (2000). Dictyostelium discoideum: a new host model system for intracellular pathogens of the genus Legionella. *Cellular Microbiology*.

[24] Isaac D. T., Laguna R. K., Valtz N., Isberg R. R. (2015). MavN is a Legionella pneumophila vacuole-associated protein required for efficient iron acquisition during intracellular growth. *Proceedings of the National Acadamy of Sciences of the United States of America*.

[25] Watanabe K., Suzuki H., Nishida T. (2018). Identification of novel Legionella genes required for endosymbiosis in Paramecium based on comparative genome analysis with Holospora spp. *FEMS Microbiology Ecology*.

[26] Jwanoswki K., Wells C., Bruce T., Rutt J., Banks T., McNealy T. L. (2017). The Legionella pneumophila GIG operon responds to gold and copper in planktonic and biofilm cultures. *PLoS ONE*.

[27] Richards A. M., Von Dwingelo J. E., Price C. T., Abu Kwaik Y. (2013). Cellular microbiology and molecular ecology of *Legionella* –amoeba interaction. *Virulence*.

[28] Murga R., Forster T. S., Brown E., Pruckler J. M., Fields B. S., Donlan R. M. (2001). Role of biofilms in the survival of *Legionella pneumophila* in a model potable-water system. *Microbiology Society*.

[29] Hamilton K. A., Prussin A. J., Ahmed W., Haas C. N. (2018). Outbreaks of legionnaires’ disease and pontiac fever 2006–2017. *Current Environmental Health Reports*.

[30] Kuroki T., Ishihara T., Ito K., Kura F. (2009). Bathwater-associated cases of legionellosis in Japan, with a special focus on Legionella concentrations in water. *Japanese Journal of Infectious Diseases*.

[31] Shakeri S., Kermanshahi R. K., Moghaddam M. M., Emtiazi G. (2007). Assessment of biofilm cell removal and killing and biocide efficacy using the microtiter plate test. *Biofouling*.

[32] Farhat M., Moletta-Denat M., Frère J., Onillon S., Trouilhé M., Robine E. (2012). Effects of disinfection on legionella spp., eukarya, and biofilms in a hot water system. *Applied and Environmental Microbiology*.

[33] Solomon J. M., Rupper A., Cardelli J. A., Isberg R. R. (2000). Intracellular growth of legionella pneumophila in dictyostelium discoideum, a system for genetic analysis of host-pathogen interactions. *Infection and Immunity*.

[34] Segal G., Shuman H. A. (1999). Legionella pneumophila utilizes the same genes to multiply within Acanthamoeba castellanii and human macrophages. *Infection and Immunity*.

[35] Ensminger A. W., Yassin Y., Miron A., Isberg R. R. (2012). Experimental evolution of Legionella pneumophila in mouse macrophages leads to strains with altered determinants of environmental survival. *PLoS Pathogens*.

